# Lenvatinib inhibits angiogenesis and tumor fibroblast growth factor signaling pathways in human hepatocellular carcinoma models

**DOI:** 10.1002/cam4.1517

**Published:** 2018-05-07

**Authors:** Masahiro Matsuki, Taisuke Hoshi, Yuji Yamamoto, Megumi Ikemori‐Kawada, Yukinori Minoshima, Yasuhiro Funahashi, Junji Matsui

**Affiliations:** ^1^ Tsukuba Research Laboratories Eisai Co., Ltd. Ibaraki Japan

**Keywords:** angiogenesis, fibroblast growth factor, hepatocellular carcinoma, Lenvatinib, vascular endothelial growth factor

## Abstract

Unresectable hepatocellular carcinoma (uHCC) is one of the most lethal and prevalent cancers worldwide, and current systemic therapeutic options for uHCC are limited. Lenvatinib, a multiple receptor tyrosine kinase inhibitor targeting vascular endothelial growth factor receptors (VEGFRs) and fibroblast growth factor receptors (FGFRs), recently demonstrated a treatment effect on overall survival by statistical confirmation of noninferiority to sorafenib in a phase 3 study of uHCC. Here, we investigated mechanisms underlying the antitumor activity of lenvatinib in preclinical HCC models. In vitro proliferation assay of nine human HCC cell lines showed that lenvatinib selectively inhibited proliferation of FGF signal‐activated HCC cells including FGF19‐expressing Hep3B2.1‐7. Lenvatinib suppressed phosphorylation of FRS2, a substrate of FGFR1–4, in these cells in a concentration‐dependent manner. Lenvatinib inhibited in vivo tumor growth in Hep3B2.1‐7 and SNU‐398 xenografts and decreased phosphorylation of FRS2 and Erk1/2 within the tumor tissues. Lenvatinib also exerted antitumor activity and potently reduced tumor microvessel density in PLC/PRF/5 xenograft model and two HCC patient‐derived xenograft models. These results suggest that lenvatinib has antitumor activity consistently across diverse HCC models, and that targeting of tumor FGF signaling pathways and anti‐angiogenic activity underlies its antitumor activity against HCC tumors.

## **I**ntroduction

Liver cancer is the second leading cause of cancer death worldwide, particularly in men 1. Hepatocellular carcinoma (HCC) is the most common type (70%–90%) of liver cancer 1. The multi‐targeted tyrosine kinase inhibitor (TKI) sorafenib, with activity against RAF kinase, vascular endothelial growth factor receptor (VEGFR)1–3, platelet‐derived growth factor receptor (PDGFR) *α* and *β*, FLT3, RET, and KIT 2, 3, was approved in 2007 and has been used as a first‐line systemic therapy for unresectable HCC (uHCC) 4. However, the clinical benefit of sorafenib is modest 5 and the five‐year relative survival of patients with uHCC remains low 4, 6. Although several TKIs have been tested versus sorafenib in clinical studies of uHCC, all the clinical trials were unsuccessful.

Lenvatinib is an orally administered, multi‐targeted TKI that selectively inhibits VEGFR1–3, fibroblast growth factor receptor (FGFR) 1–4, PDGFR *α*, RET, and KIT 3, 7. Preclinical studies have revealed that lenvatinib potently blocks VEGF‐ and FGF‐driven angiogenesis, KIT‐dependent angiogenesis, RET‐fusion/RET mutant tumorigenesis, and VEGFR3‐associated lymphangiogenesis 3, 7, 8, 9, 10, 11. Lenvatinib has been used to treat progressive, locally recurrent or metastatic, radioactive iodine‐refractory differentiated thyroid cancer (USA and EU), and unresectable thyroid cancer (Japan) 12. In addition, lenvatinib in combination with everolimus is approved for the treatment of metastatic renal cell carcinoma following VEGF‐targeted therapy 13. In a recent phase 3 clinical trial of lenvatinib for patients with uHCC (NCT01761266), lenvatinib demonstrated a treatment effect on overall survival by statistical confirmation of noninferiority to sorafenib and achieved significant and clinically meaningful improvement in objective response rate, progression‐free survival, and time to progression 14.

Genomic analysis of HCC has provided insight into the driver genes or pathways 15, 16. For instance, a focal amplification on chromosome 11q13 (*FGF19* and *CCND1*) and overexpression of the *FGF19* gene has been identified in a subset of HCCs, and multiple studies have demonstrated the roles of the FGF19–FGFR4 axis in HCC 17, 18, 19, 20. In addition, other studies of HCC preclinical models have shown the importance of FGF signal pathways 21, 22, 23. Therefore, targeting of FGFR signaling in HCC is attracting a great of attention.

Here, we investigated the antitumor activity of lenvatinib in various preclinical HCC models and explored the mechanisms underlying its antitumor activity.

## Materials and Methods

### Cell lines and reagents

Hep3B2.1‐7, SNU‐398, and SNU‐449 cells were obtained from the American Type Culture Collection (Manassas, VA); HuH‐1, HuH‐7, and JHH‐7 24 cells were from Japanese Collection of Research Bioresources Cell Bank (Osaka, Japan); Li‐7 and PLC/PRF/5 cells were from the Cell Resource Center for Biomedical Research, Institute of Development, Aging and Cancer, Tohoku University (Miyagi, Japan); and SK‐HEP‐1 cells were from DS Pharma Biomedical (Tokyo, Japan). Lenvatinib (lenvatinib mesilate) was synthesized at Eisai Co., Ltd. (Ibaraki, Japan). Sorafenib (sorafenib tosylate) was prepared from sorafenib tablets (Bayer Yakuhin, Ltd., Osaka, Japan). Recombinant human FGF19 was obtained from R&D Systems (Minneapolis, MN).

### Cell culture

Cell lines were incubated at 37°C in 5% CO_2_ in the following media containing 10% FBS and 100 units/mL penicillin–100 *μ*g/mL streptomycin: EMEM (Wako, Osaka, Japan) for Hep3B2.1‐7; DMEM (Wako) for HuH‐7; Williams’ Medium E (Sigma–Aldrich, St. Louis, MO) for JHH‐7; high‐glucose DMEM (Wako) for SK‐HEP‐1, HuH‐1, and PLC/PRF/5; RPMI‐1640 (Wako) for Li‐7, SNU‐398, and SNU‐449; and RPMI‐1640 (Invitrogen, Carlsbad, CA) supplemented with 10 *μ*g/mL insulin and 2 *μ*mol/L hydrocortisone, for LIXC‐012.

### HCC xenograft models

Animal experiments were performed in accordance with the guidelines approved by the Institutional Animal Care and Use Committee of Eisai Co., Ltd., Shanghai ChemPartner Co., Ltd., or Crown Bioscience Inc. Lenvatinib was dissolved in 3 mmol/L HCl. Sorafenib was dissolved in cremophor:ethanol (1:1) and then diluted fourfold with distilled water. HCC cells (2.5 × 10^6^ to 5 × 10^6^) in HBSS (for Hep3B2.1‐7 and SNU‐398) or culture medium (for PLC/PRF/5) were mixed with Matrigel [1:1] (Corning, Corning, NY) and then inoculated subcutaneously into the right flank of female BALB/c nude mice. When the tumors reached approximately 170–290 mm^3^, the mice were randomly allocated to treatment groups (Day 1). Lenvatinib, sorafenib, or the corresponding vehicle was given orally to individual mice once daily for the indicated doses and periods in each figure legend.

The LIXC‐012 xenograft study was performed at Shanghai ChemPartner Co., Ltd. (Shanghai, China). LIXC‐012 cells (2 × 10^6^) in serum‐free medium mixed with Matrigel [1:1] (Corning) were inoculated subcutaneously into the right flank of female nu/nu mice. When the tumors reached approximately 80–300 mm^3^, the mice were randomly allocated to treatment groups (Day 1); mice then orally received lenvatinib, sorafenib, or the corresponding vehicle once daily for 14 days. Multiple mice in each vehicle control group were removed from the study because of excess tumor volume or cachexia‐induced body weight loss (BWL) after Day 11.

Patient‐derived xenograft (PDX; LI0050 and LI0334) studies were performed at Crown Bioscience Inc. (Taicang, China). Each tumor fragment (diameter, 2–3 mm) was inoculated subcutaneously into the right flank of female BALB/c nude mice. When the tumors reached approximately 130–150 mm^3^ (Day 1), the mice were randomly allocated to treatment groups; mice then orally received lenvatinib, sorafenib, or vehicle (3 mmol/L HCl only) once daily for 28 days. In the PDX studies, when BWL was ≥20% for any individual mouse, that mouse was given dosing holiday(s) until its body weight recovered to baseline (BWL, ≤10%).

Tumor dimensions were measured two or three times weekly with a caliper, and tumor volume was calculated as ½ × length × width^2^. Relative body weight (RBW) was calculated by dividing the daily body weight by that of the same mouse on Day 1.

### Western blot analysis

Cells were washed and lysed with RIPA buffer containing protease inhibitor cocktail (Roche Diagnostics, Mannheim, Germany) and Halt phosphatase inhibitor cocktail (Thermo Fisher Scientific, Waltham, MA). To examine the effect on FGF signaling pathways in vitro, HCC cells were treated with lenvatinib, sorafenib, or vehicle (DMSO) for 1 h before cell lysis. In the case of Hep3B2.1‐7, the cells were starved overnight in culture medium containing 0.5% BSA; incubated with each drug for 1 h; treated with 100 ng/mL FGF19 for 5 min; and then washed and lysed. To examine the inhibitory effect on FGF signaling pathways in vivo, mice bearing xenograft tumors underwent single treatment with lenvatinib, sorafenib, or the corresponding vehicle; the tumors were collected 2 h later; and the samples were homogenized and lysed.

Cell and tumor lysates were cleared by centrifugation, denatured in 1× Laemmli sample buffer, separated by SDS‐PAGE, and transferred to PVDF membranes. The membranes were incubated with antibodies to FGFR1, FGFR3, FGFR4, p‐FRS2 (Tyr436), Erk1/2, and p‐Erk1/2 (Cell Signaling Technology, Beverly, MA), FGFR2 [Bek] (Santa Cruz Biotechnology, Dallas, TX), KLB (*β*‐Klotho) and FRS2 (R&D Systems), *β*‐actin and FGF19 (Sigma‐Aldrich) at the recommended concentrations. Bands were visualized as described previously 25.

### In vitro proliferation assay

Human HCC cells were plated on 96‐well plates at 1 × 10^3^ cells/well and cultured at 37°C in 5% CO_2_. Next day, the cells were treated with 1:2 serial dilutions of lenvatinib, sorafenib, or vehicle (DMSO), and cultured for 6 days. Cell viability was measured by using a Cell Counting Kit‐8 (Dojindo Molecular Technologies, Kumamoto, Japan), and IC_50_ values were determined using GraphPad Prism (GraphPad Software, La Jolla, CA). Each experiment contained triplicate samples, and three independent experiments were performed.

### Immunohistochemistry

Xenograft tumors were collected on the day after the last dosing; tumor fragments were fixed with 10% formalin and embedded in paraffin, except for LI0334 tumor fragments, which were frozen in OCT compound and the tissue sections were fixed with cold acetone. Staining for endothelial cells with anti‐CD31 antibody and microvessel density (MVD) measurement were performed as described previously 25. Briefly, tissue sections were stained with anti‐CD31 antibody (Dianova, Hamburg, Germany) and the slides were scanned using the Aprio ScanScope XT system. Five regions of interest (ROIs, each 500 × 500 *μ*m) with the highest densities of CD31‐stained microvessels were selected manually, and the number of microvessels in each ROI was measured.

Ki‐67‐positive cells were stained with anti‐Ki‐67 antibody (Cell Signaling Technology) and detected using Envision+ Single Reagents (Dako, Agilent Technologies, Santa Clara, CA). The percentage of Ki‐67 positive nuclei was quantified using HALO software (Indica Labs, Corrales, NM).

### Statistical analysis

Unless otherwise indicated, tumor volume, MVD, and Ki‐67‐positive cells (%) were compared between treatment groups using the Dunnett's multiple comparison test. *P* values <0.05 were considered significant.

Additional Materials and Methods are provided in Appendix S1 and Tables S1–S3.

## Results

### Antiproliferative activity of lenvatinib against HCC cell lines

**Figure 1:**
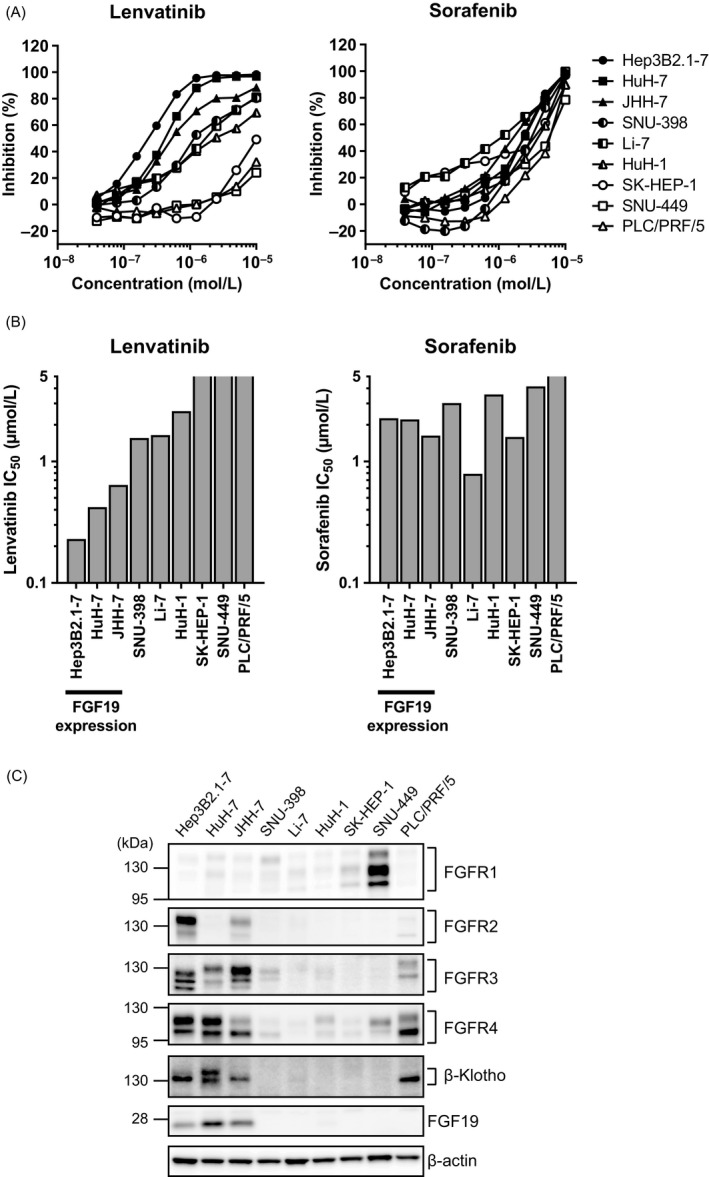
In vitro antiproliferative activity of lenvatinib and sorafenib in nine human HCC cell lines. (A) Inhibition of HCC cell proliferation by lenvatinib and sorafenib. Data are presented as means of three independent experiments performed in triplicate. (B) IC_50_ values for HCC cells, placed in the order of ascending values for lenvatinib. (C) Expression levels of FGF19, FGFR1–4, and *β*‐Klotho, as determined by Western blot analysis. Multiple bands represent different isoforms and/or post‐translational modifications of FGFRs and *β*‐Klotho.

Activation of the FGF signaling pathways is associated with HCC progression, and proliferation of subsets of HCC is dependent on FGF signaling pathways 17, 19, 20, 21, 23, 26. Because lenvatinib inhibits FGFRs, we first examined the antiproliferative activity of lenvatinib against nine HCC cell lines in vitro. Lenvatinib showed selective and potent antiproliferative activity against the HCC cell lines Hep3B2.1‐7, HuH‐7, and JHH‐7, with IC_50_ values of 0.23, 0.42, and 0.64 *μ*mol/L, respectively (Fig. 1A and B). Immunoblotting analysis showed that FGF19, FGFR4, and *β*‐Klotho were highly expressed in these three cell lines, indicating that the FGF19–FGFR4 axis was activated (Fig. 1C), consistent with previous reports 20, 26. Lenvatinib also showed moderate inhibitory effects on the proliferation of SNU‐398, Li‐7, and HuH‐1 cells, with IC_50_ values of 1.56, 1.65, and 2.59 *μ*mol/L, respectively (Fig. 1A and B). Although FGF19 protein was not expressed, some members of the FGFR family were detected in these cells (Fig. 1C). Because these cell lines are modestly sensitive to pan‐FGFR inhibitors such as BGJ398 20, 21, 26, lenvatinib might inhibit proliferation of the cells by targeting an activated FGF signaling pathway. Lenvatinib did not show clear antiproliferative activity against SK‐HEP‐1, SNU‐449, or PLC/PRF/5 cells (IC_50_ >5 *μ*mol/L; Fig. 1A and B), which were also insensitive to pan‐FGFR inhibitors 20, 26.

As a reference, we also examined the antiproliferative activity of sorafenib, an approved systemic treatment drug for uHCC 4, 27, in the nine HCC cell lines. Sorafenib did not show selective antiproliferative activity against HCC cell lines with high FGF19 expression (Fig. 1). These results indicate that lenvatinib selectively suppressed the proliferation of HCC cells with activated FGF signaling pathways.

Lenvatinib binds to the receptor tyrosine kinase VEGFR2 with Type V binding mode 28; this may contribute to its high affinity and its selectivity for a small number of receptor tyrosine kinases. Co‐crystal analysis with lenvatinib–FGFR1 complex revealed that lenvatinib also bound to FGFR1 with Type V binding mode (Fig. S1). Homology modeling and docking simulation based on the lenvatinib–FGFR1 complex suggest that the mode of binding to FGFR2, 3, and 4 is identical to that to FGFR1 (Figs S2 and S3).

### Inhibitory effect of lenvatinib on FGF signaling pathways in HCC cell lines

**Figure 2:**
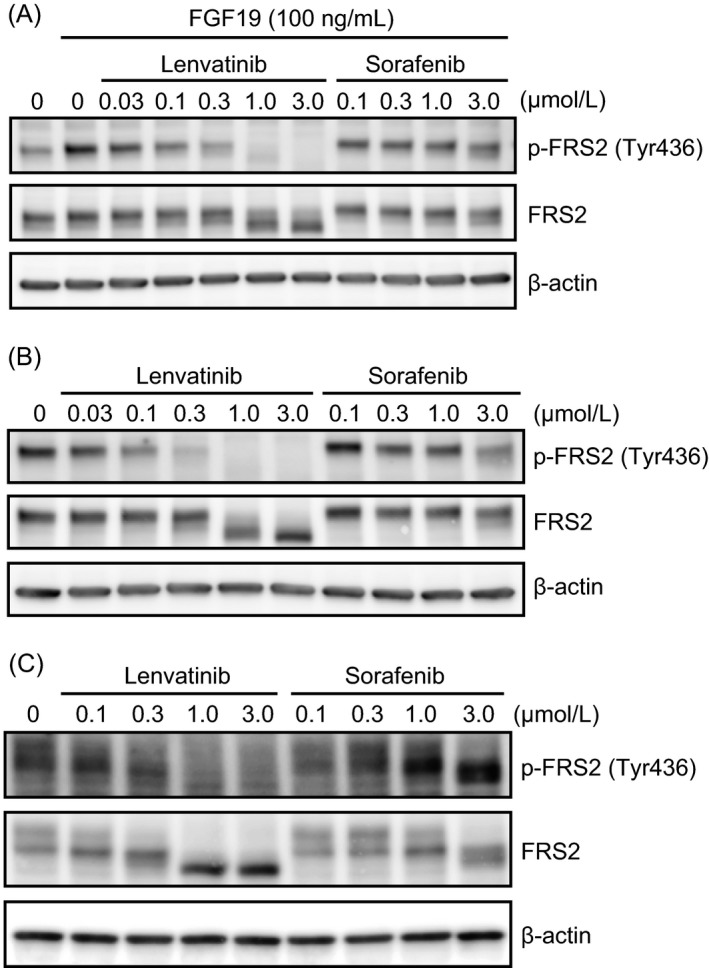
Inhibitory effects of lenvatinib and sorafenib on FGF signaling pathways in human HCC cells. (A) Hep3B2.1‐7 cells were treated with lenvatinib, sorafenib, or vehicle (DMSO) for 1 h, followed by stimulation with FGF19 for 5 min. (B) HuH‐7 cells and (C) SNU‐398 cells were treated with each drug or vehicle for 1 h. Phosphorylated FRS2 (p‐FRS2), FRS2, and *β*‐actin were detected by Western blot analysis.

We next examined the effects of lenvatinib on FGF signaling pathways in the HCC cell lines by Western blot analysis. In Hep3B2.1‐7 and HuH‐7 cells, which are highly expressing FGF19, constitutive phosphorylation of FRS2, an FGFR downstream signaling molecule, was observed; in Hep3B2.1‐7 cells, this phosphorylation was further enhanced by the addition of recombinant human FGF19. Lenvatinib suppressed the phosphorylation of FRS2 at 0.03–3 *μ*mol/L in both cell types (Fig. 2A and B). The IC_50_ values of lenvatinib in the proliferation assay against these cells were parallel to the concentrations of lenvatinib for pFRS2 inhibition. Furthermore, lenvatinib dose dependently inhibited FRS2 phosphorylation in SNU‐398 cells (Fig. 2C). Lenvatinib inhibited the basic FGF (bFGF)‐stimulated phosphorylation of FRS2 in SNU‐449 cells (Fig. S4), which express high levels of FGFR1, although lenvatinib did not show potent antiproliferative activity against the cells (Fig. 1). In contrast, sorafenib did not show clear inhibition of FRS2 phosphorylation among the HCC cell lines tested. These results suggest that lenvatinib‐mediated blockade of FGFRs in HCC cells with activated FGF signaling pathways is the likely basis of the antiproliferative activity of this compound in vitro.

### Antitumor activity of lenvatinib against xenograft models with activated FGF signaling pathways

**Figure 3:**
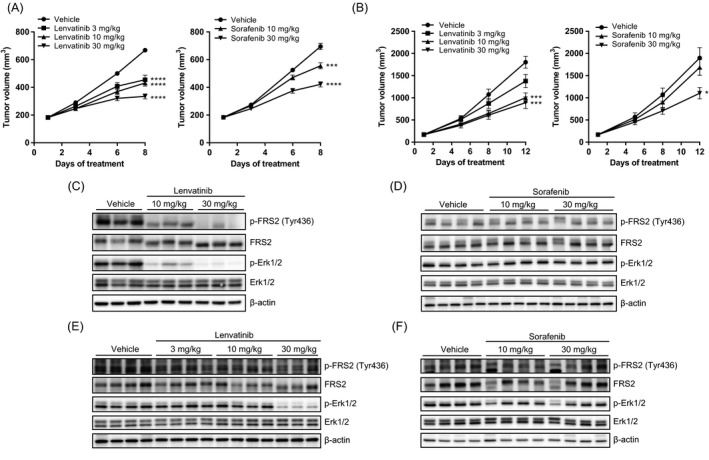
Effects of lenvatinib and sorafenib in human HCC xenograft models. Mice bearing xenograft tumors were orally administered lenvatinib (3–30 mg/kg), sorafenib (10, 30 mg/kg), or the corresponding vehicle for 7 (Hep3B2.1‐7) or 11 (SNU‐398) days. (A, B) Antitumor activity in the Hep3B2.1‐7 (A) and SNU‐398 (B) xenograft models. Data are means ± SEM (*n* = 8) *P <* *0.05, ***P <* *0.001, ****P <* *0.0001 versus vehicle control. (C–F) Inhibitory activity against phosphorylation of FRS2 and Erk1/2 in the xenograft‐derived tumors. Tumors were collected 2 h after single treatment with lenvatinib (C, E) or sorafenib (D, F) at the indicated doses. Lysates of Hep3B2.1‐7 tumors (C, D) and SNU‐398 tumors (E, F) were then subjected to Western blot analysis.

We next evaluated the in vivo antitumor activity of lenvatinib against HCC xenografts with activated FGF signaling pathways in the Hep3B2.1‐7 and SNU‐398 models. In vivo growth of xenograft tumors was significantly inhibited by lenvatinib at doses of 3–30 mg/kg in the Hep3B2.1‐7 model and 10, 30 mg/kg in the SNU‐398 model, and by sorafenib at 10 and 30 mg/kg in the Hep3B2.1‐7 model and 30 mg/kg in the SNU‐398 model (Fig. 3A and B). BWL of the treatment groups was similar to that of the corresponding vehicle control group, although cachexia‐induced BWL was observed in each control group (Fig. S5).

To examine whether lenvatinib inhibited FGF signaling pathways within the Hep3B2.1‐7 and SNU‐398 xenograft tumors, tumor samples were collected 2 h after a single lenvatinib treatment, and phosphorylation levels of FRS2 and another downstream molecule of FGFR, Erk1/2, were evaluated. Lenvatinib at 10 and 30 mg/kg inhibited phosphorylation of FRS2 and Erk1/2 in the Hep3B2.1‐7 model (Fig. 3C); lenvatinib at 3–30 mg/kg inhibited FRS2 phosphorylation in the SNU‐398 model (Fig. 3E); and lenvatinib at 30 mg/kg inhibited Erk1/2 phosphorylation in the SNU‐398 model (Fig. 3E). In contrast, no clear suppression was observed in sorafenib‐treated xenograft tumors in either model (Fig. 3D and F). Inhibition of the FGF signaling pathway in vivo was also observed in human HCC HuH‐7 xenografts, which overexpressed FGF19 (Fig. S6). These results suggest that targeting FGFR of HCC cells underlies the antitumor activity of lenvatinib in preclinical HCC models with activated FGF signaling pathways.

### Antitumor activity of lenvatinib against PLC/PRF/5 xenograft model

**Figure 4:**
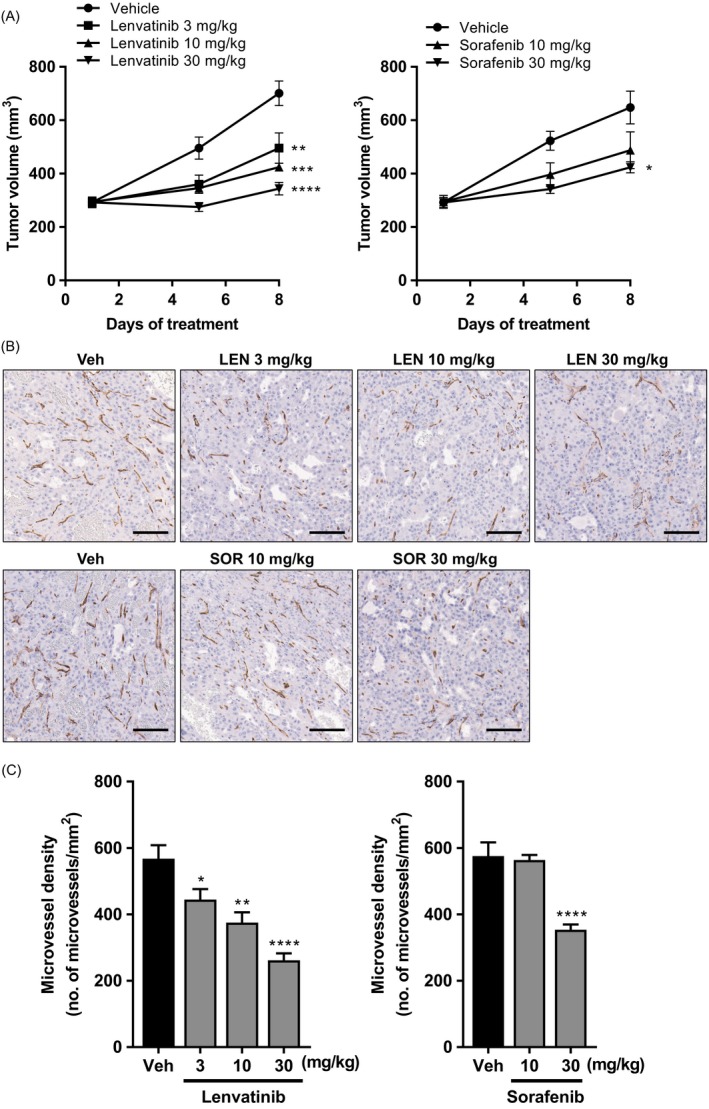
Antitumor and anti‐angiogenic activities of lenvatinib and sorafenib in the PLC/PRF/5 xenograft model. Mice bearing xenograft tumors were orally administered lenvatinib (LEN; 3–30 mg/kg), sorafenib (SOR; 10, 30 mg/kg), or the corresponding vehicle (Veh) for 7 days. (A) Tumor growth curves of PLC/PRF/5 xenograft model. Data are means ± SEM (*n* = 6). (B, C) Formalin‐fixed paraffin‐embedded sections of tumors collected on Day 8 were stained with anti‐CD31 antibody to visualize microvessels in the tumors. (B) Representative images of the tumor microvessels. Bar = 100 *μ*m. (C) Quantification of MVD. Data are presented as means + SEM (*n* = 6). *P <* *0.05, **P <* *0.01, ***P <* *0.001, ****P <* *0.0001 versus vehicle control.

In contrast to Hep3B2.1‐7, HuH‐7, and SNU‐398 cells, PLC/PRF/5 cells were not sensitive to lenvatinib and other FGFR inhibitors in vitro 20, 26 and do not overexpress FGF19, even though FGFR4 is expressed (Fig. 1), suggesting that proliferation of PLC/PRF/5 cells was not dependent on an activated FGF signaling pathway. Lenvatinib inhibited in vivo tumor growth of PLC/PRF/5 xenografts at all doses tested (Fig. 4A), and sorafenib also inhibited in vivo tumor growth at 30 mg/kg (Fig. 4A). No obvious BWL was noted in either treatment group compared with its corresponding control group (Fig. S7). In an additional experiment, the maximum antitumor activity of lenvatinib was greater than that of sorafenib in the PLC/PRF/5 model (Fig. S8). To examine whether the antitumor activity of lenvatinib and sorafenib against PLC/PRF/5 xenografts was based on angiogenesis inhibition, we measured MVD within PLC/PRF/5 xenograft tumors. In accordance with the antitumor activity, tumor MVD was significantly decreased by Day 8 following lenvatinib treatment at 3–30 mg/kg and sorafenib treatment at 30 mg/kg (Fig. 4B and C). These results suggest that lenvatinib can exert antitumor activity against HCC tumors whose proliferation is not dependent on an FGF signaling pathway, via its anti‐angiogenic effect.

We also measured MVD in the Hep3B2.1‐7 and SNU‐398 xenograft models after treatment with lenvatinib and sorafenib. Lenvatinib (3–30 mg/kg for Hep3B2.1‐7 model; 10 and 30 mg/kg for SNU‐398 model) and sorafenib (30 mg/kg for both models) significantly reduced MVD in xenograft tumors (Fig. S9), suggesting that lenvatinib could target the FGFR of HCC cells and tumor angiogenesis in the preclinical HCC models with activated FGF signaling pathways, whereas sorafenib targeted mainly tumor angiogenesis or an undefined signaling pathway.

### Antitumor activity of lenvatinib in a xenograft model of patient‐derived xenograft (PDX)‐derived cell line and two HCC PDX models

**Figure 5:**
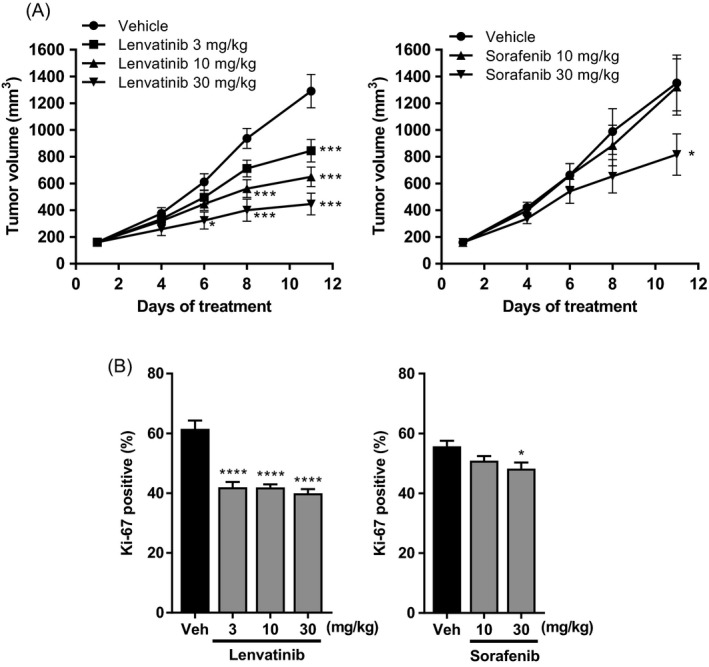
Effects of lenvatinib and sorafenib in the HCC PDX‐derived cell line (LIXC‐012) xenograft model. Mice bearing LIXC‐012 tumors were orally administered lenvatinib (3–30 mg/kg), sorafenib (10, 30 mg/kg), or the corresponding vehicle (Veh). Formalin‐fixed paraffin‐embedded sections of tumors collected the day after the last dosing were stained with anti‐Ki‐67 antibody to visualize proliferating tumor cells. (A) Tumor growth curves of the LIXC‐012 xenograft model. Data are means ± SEM (*n* = 8). *P <* *0.05, ***P <* *0.001 versus vehicle control on Days 1–11 (two‐way analysis of variance followed by Bonferroni's multiple comparison test). (B) Numbers of Ki‐67 positive cells (%). Data are means + SEM (*n* = 7 [Veh for lenvatinib] to 8). *P <* *0.05, ****P <* *0.0001 versus vehicle control.

We examined the antitumor activity of lenvatinib using a xenograft model of PDX‐derived cell line and two HCC PDX models. PDX LIX‐012 robustly expresses *FGF19*, although it does not possess *FGF19* gene amplification 20. The LIXC‐012 cell line, which was established from PDX LIX‐012 29, overexpresses *FGF19, FGFR4*, and *KLB* and is sensitive to BLU9931, a selective FGFR4 inhibitor, suggesting that FGF19‐activated FGF signaling plays a role in LIXC‐012 models 20. Here, lenvatinib at 3–30 mg/kg and sorafenib at 30 mg/kg inhibited the in vivo growth of LIXC‐012 xenograft tumors (Fig. 5A). Cachexia‐induced BWL was observed in this model, but mice treated with lenvatinib at 10 and 30 mg/kg showed recovery of BWL (Fig. S10). To examine whether lenvatinib inhibits LIXC‐012 cell proliferation in vivo, we stained for the proliferation marker Ki‐67 within xenograft tumors collected 1 day after the last lenvatinib dose. Lenvatinib treatment at 3–30 mg/kg significantly reduced the number of Ki‐67‐positive cells (Fig. 5B). A significant but modest inhibition of Ki‐67 staining was observed with sorafenib treatment at 30 mg/kg only (Fig. 5B).

**Figure 6:**
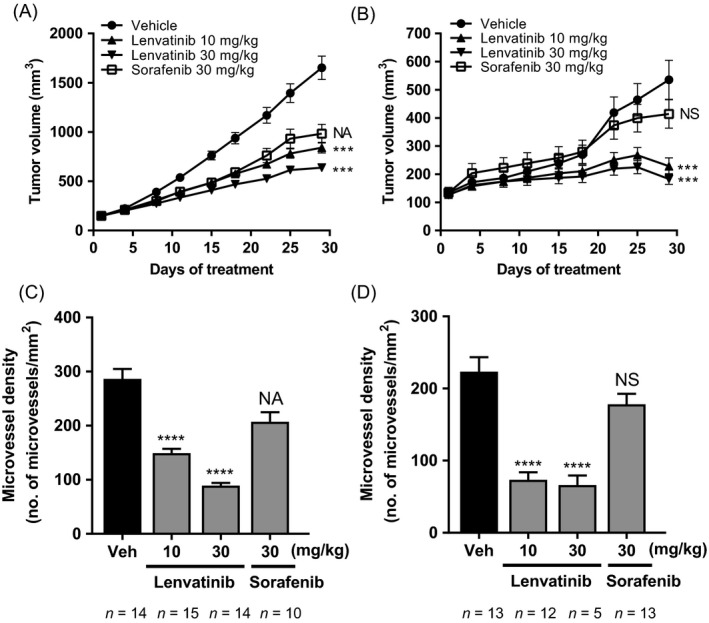
Antitumor and anti‐angiogenic activities of lenvatinib and sorafenib in two HCC PDX models. Mice bearing tumors were orally administered lenvatinib (10, 30 mg/kg), sorafenib (30 mg/kg), or vehicle (Veh; 3 mmol/L HCl only) for 28 days. (A) Tumor growth curve of LI0050 model. In this study sorafenib was poorly tolerated, with the death of five mice (one on Day 10 and four on Day 19) and multiple dose suspensions, and 30 mg/kg lenvatinib treatment of one mouse was suspended for 13 days owing to transient BWL on Day 15. Data are means ± SEM;* n* = 15 (vehicle control and lenvatinib groups) or *n* = 10–15 (sorafenib group). NA, not applicable. (B) Tumor growth curve of LI0334 model. Data are means ± SEM (*n* = 15). NS, not significant. (C, D) MVD in the LI0050 (C) and LI0334 (D) models. Data are means + SEM. ***P <* *0.001, ****P <* *0.0001 versus vehicle control.

We also examined the inhibitory effect of lenvatinib on tumor growth in two HCC PDX models, LI0050 and LI0334. Lenvatinib at 10 and 30 mg/kg inhibited the tumor growth of both PDXs, and this was accompanied by a dramatic and significant decrease in MVD (Fig. 6). Sorafenib at 30 mg/kg was not tolerated in the LI0050 model and failed to show antitumor and anti‐angiogenic activities in the LI0334 model (Fig. 6). In both models, BWL in the lenvatinib treatment groups was comparable to that in the vehicle control group (Fig. S11). These results indicate that lenvatinib had antitumor activity against HCC PDX models, likely through its potent anti‐angiogenic activity. Collectively, the antitumor activity of lenvatinib was more robust than that of sorafenib in a wide variety of preclinical HCC models, including cell line xenograft and PDX models (Fig. S12).

## Discussion

Lenvatinib is an inhibitor of multiple tyrosine kinases including VEGFRs and FGFRs 3, 7. In particular, the potent activity against FGFR1–4 is a distinctive feature of lenvatinib, compared with sorafenib. In addition to kinase profile, the binding mode with target kinases differs between the two drugs: lenvatinib binds to VEGFR2 in a Type V binding mode, whereas sorafenib has a Type II binding mode 28, 30. Here, we discovered that lenvatinib also binds to FGFR1 in a Type V binding mode, and homology modeling and docking simulations suggest that the mode of binding to FGFR2, FGFR3, and FGFR4 is identical to that to FGFR1 (Figs S1–S3). Thus, these differences in molecular characteristics of lenvatinib and sorafenib may underlie the differences in the pharmacological activities and mechanisms of action of these drugs.

Increasing evidence suggests that activation of FGF signaling pathways in HCC contributes to its malignancy 17, 19, 20, 21, 23, 26. Therefore, we examined the antiproliferative activity of lenvatinib against nine HCC cell lines, and we showed that lenvatinib has inhibitory activity against HCC cell lines with activated FGF signaling pathways 20, 26; this proliferation inhibition was accompanied by suppressed FRS2 phosphorylation (Figs 1 and 2). These results indicate that the selective antiproliferative activity of lenvatinib was based on inhibition of activated FGF signaling pathways. Inhibition of FGF signaling by lenvatinib was also observed in xenograft tumors (Fig. 3). Further study is warranted to investigate how an inhibitory effect of lenvatinib on tumor FGF signaling pathways contributes to its antitumor activity in the HCC models. Sorafenib has inhibitory activity against RAF kinase 2, 27 acting downstream of receptor tyrosine kinases and upstream of ErK1/2. However, in the current study, sorafenib showed neither selectivity against the proliferation of HCC cell lines with the activated FGF pathway nor downregulation of p‐Erk1/2 in xenograft tumors (Figs 1 and 3), suggesting that sorafenib may not affect FGF signaling pathways in these preclinical HCC models.

Autocrine activation of FGF signaling involving FGFR4 and its cognate ligand FGF19 is reported in about one‐third to one‐half of HCC patients 31, 32, 33, 34. Therefore, targeting FGFR4 is expected to be an effective approach for treatment of uHCC. Indeed, a selective FGFR4 inhibitor has recently been shown in a phase 1 clinical trial of patients with advanced HCC to provide objective clinical activity in FGF19‐positive disease 35. Here, we showed that lenvatinib inhibited tumor growth and suppressed the FGF signaling pathway in FGF19‐overexpressing HCC xenograft models. In contrast, sorafenib did not affect the FGF signaling pathway in vitro and in vivo, suggesting that the mode of actions of the two drugs differs in HCC models with FGF19 overexpression.

In addition to the FGF19–FGFR4 axis, another FGF–FGFR axis might affect cell proliferation and in vivo tumor growth of HCC xenograft tumors 21, 22, 23. In preclinical experiments using HCC PDX models, lenvatinib had a greater response than sorafenib in those models highly expressing *FGFR1* mRNA 36. In our study, lenvatinib inhibited the proliferation of SNU‐398, Li‐7, and HuH‐1 cells in vitro (Fig. 1A and B), all of which did not produce FGF19 (Fig. 1C) but are known to be sensitive to FGFR inhibitors 20, 21, 26. Given that these HCC cell lines expressed multiple FGFRs (Fig. 1C), further experiments are required to delineate the FGFR(s) responsible for tumor growth. Most importantly, our results suggest that lenvatinib can have a direct antiproliferative effect on HCC cells with an activated FGF signaling pathway that does not involve the FGF19–FGFR4 axis. Long‐term exposure to sorafenib confers sorafenib resistance on HCC cells with upregulation of FGFR1 37. Considering this result, lenvatinib has the potential to overcome such resistance by blocking FGFRs.

Because HCCs are highly angiogenic, anti‐angiogenic therapy is a promising approach to the treatment of uHCC 38, 39, 40. Here, lenvatinib inhibited tumor angiogenesis in all HCC xenograft models tested, regardless of the activation status of FGF signaling pathways, suggesting that inhibition of angiogenesis underlies the antitumor activity of lenvatinib in HCC models. The anti‐angiogenic activity of lenvatinib was more potent than that of sorafenib, especially in PDX models (Fig. 6). Accumulating evidence indicates that FGF acts as a pro‐angiogenic factor and induces escape from anti‐VEGF therapy 41. Consistently, anti‐bFGF monoclonal antibody inhibits angiogenesis and has additive effects in combination with sorafenib in HCC xenograft models 42. We previously revealed that lenvatinib targets VEGF‐ and FGF‐induced angiogenesis 7, 8, 11, and therefore the blocking of not only VEGFR but also FGFR by lenvatinib might be behind its potent anti‐angiogenic and antitumor activities in the HCC models.

Recently, an anti‐programmed death‐1 (PD‐1) antibody, nivolumab, provided clinically meaningful responses in patients with uHCC in a phase 1/2 study (NCT01658878), suggesting that tumor immune regulation also plays an important role in the antitumor effect in uHCC 43. Interestingly, VEGF modulates immune responses and targeting VEGF improved the activity of anti‐PD‐1 treatment in preclinical models 44, 45. In addition, FGFR3 activation is associated with low T‐cell infiltration in bladder cancer, and the FGF signaling pathway participates in tumor immunity 46, 47. Considering these findings, further studies are crucial to elucidate the effect of lenvatinib‐mediated dual blockade of VEGFR and FGFR on tumor immunity in HCC models. These studies may provide insights into the antitumor activity and molecular mechanisms of lenvatinib in the clinical setting.

In conclusion, we demonstrated that lenvatinib exerts robust antitumor activity against various HCC models, including cell line xenograft and PDX models, and that blockade of activated FGF signaling pathways in HCC tumor cells and potent anti‐angiogenic activity underlie these antitumor activities.

## Conflict of Interest

All authors are employees of Eisai Co., Ltd.

## Supporting information


**Figure S1.** X‐ray analysis of crystal structure of FGFR1–lenvatinib complex.
**Figure S2.** Sequence alignment of FGFR1–4, secondary structural elements of FGFR1, and residue positions with shortest distance to lenvatinib.
**Figure S3.** Docking models of FGFR2–4 with lenvatinib.
**Figure S4.** Inhibitory activity of lenvatinib and sorafenib against the FGF signaling pathway in SNU‐449 cells.
**Figure S5.** Relative body weight of mice bearing Hep3B2.1‐7 or SNU‐398 xenografts with lenvatinib or sorafenib treatment.
**Figure S6.** Inhibitory activity of lenvatinib and sorafenib against the FGF signaling pathway in HuH‐7 xenograft tumors.
**Figure S7.** Relative body weight of mice bearing PLC/PRF/5 xenografts with lenvatinib or sorafenib treatment.
**Figure S8.** Antitumor activity of lenvatinib and sorafenib in the PLC/PRF/5 xenograft model.
**Figure S9.** Anti‐angiogenic activities of lenvatinib and sorafenib in Hep3B2.1‐7 and SNU‐398 xenograft models.
**Figure S10.** Relative body weight of mice bearing PDX‐derived cell line LIXC‐012 xenografts with lenvatinib or sorafenib treatment.
**Figure S11.** Relative body weight of mice bearing HCC PDX (LI0050 or LI0334) tumors with lenvatinib or sorafenib treatment.
**Figure S12.** Summary of tumor growth inhibition (TGI) in HCC xenograft models.

**Table S1.** Data collection and processing statistics.
**Table S2.** Refinement statistics.
**Table S3.** Glide docking score.

**Appendix S1.** Supporting materials and methods.
