# Supplementary material for: Lenvatinib inhibits angiogenesis and tumor fibroblast growth factor signaling pathways in human hepatocellular carcinoma models
Source: Cancer Med. 2018 May 7;7(6):2641–53. doi: 10.1002/cam4.1517 (PMC6010799; doi:10.1002/cam4.1517)
Supplement: Supplementary file 1 — Figure S1. X‐ray analysis of crystal structure of FGFR1–lenvatinib complex. Figure S2. Sequence alignment of FGFR1–4, secondary structural elements of FGFR1, and residue positions with shortest distance to lenvatinib. Figure S3. Docking models of FGFR2–4 with lenvatinib. Figure S4. Inhibitory activity of lenvatinib and sorafenib against the FGF signaling pathway in SNU‐449 cells. Figure S5. Relative body weight of mice bearing Hep3B2.1‐7 or SNU‐398 xenografts with lenvatinib or sorafenib treatment. Figure S6. Inhibitory activity of lenvatinib and sorafenib against the FGF signaling pathway in HuH‐7 xenograft tumors. Figure S7. Relative body weight of mice bearing PLC/PRF/5 xenografts with lenvatinib or sorafenib treatment. Figure S8. Antitumor activity of lenvatinib and sorafenib in the PLC/PRF/5 xenograft model. Figure S9. Anti‐angiogenic activities of lenvatinib and sorafenib in Hep3B2.1‐7 and SNU‐398 xenograft models. Figure S10. Relative body weight of mice bearing PDX‐derived cell line LIXC‐012 xenografts with lenvatinib or sorafenib treatment. Figure S11. Relative body weight of mice bearing HCC PDX (LI0050 or LI0334) tumors with lenvatinib or sorafenib treatment. Figure S12. Summary of tumor growth inhibition (TGI) in HCC xenograft models. [file CAM4-7-2641-s001.pdf]

Supporting figures

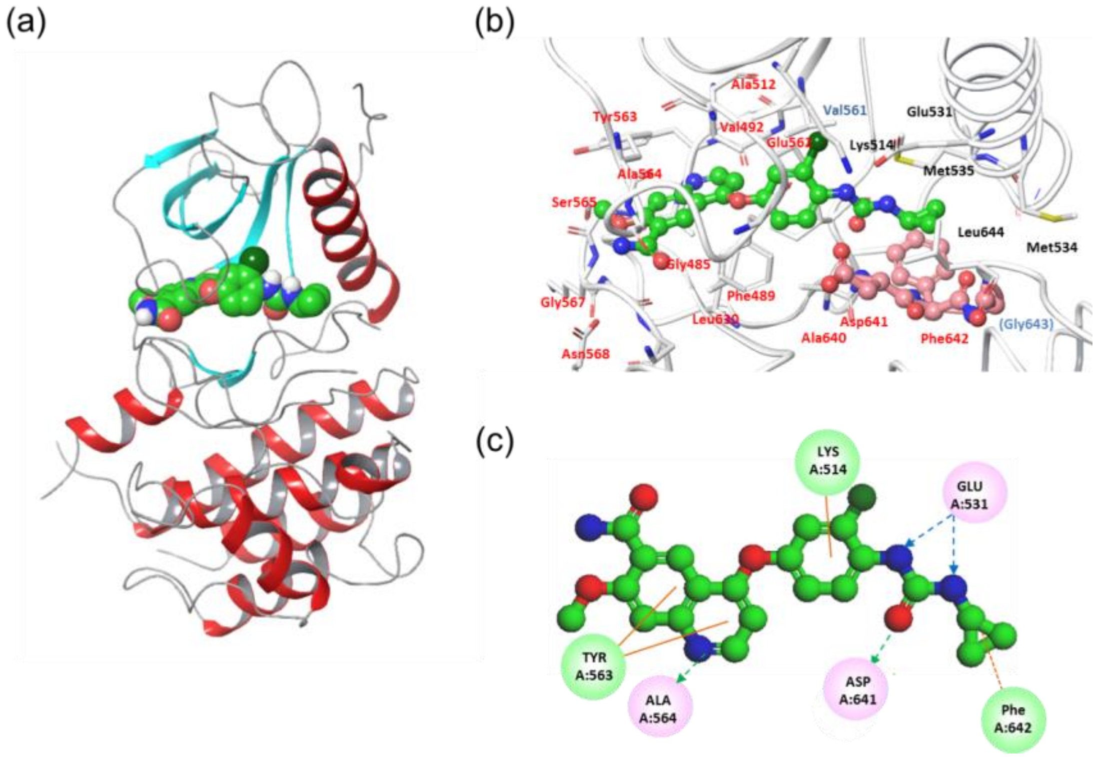

Fig. S1. X-ray analysis of crystal structure of FGFR1–lenvatinib complex.

(a) Overall structure of the FGFR1–lenvatinib complex. Lenvatinib is shown as a CPK model colored according to chemical atom type, with carbon atoms shown as green. One monomer of FGFR1 is represented as a ribbon. The beta-sheet, helixes, and other components of the FGFR1 are shown in blue, red, and gray, respectively. (b) Binding pocket of FGFR1 complexes. Lenvatinib and neighboring protein side chains are shown as stick models colored according to the chemical atom type, as described above. Carbon atoms of the DFG (Asp-Phe-Gly) domain are salmon colored. Residue names at ATP-binding sites are indicated by red letters, and neighboring regions by black letters. The gatekeeper residue, Val561, is indicated by blue. (c) Scheme of interaction between FGFR1 amino acid residues and lenvatinib. Residues that interact with lenvatinib are circled by a different color according to the type of interaction: those involved in hydrogen bonding, charge, or polar interactions are in pink circles; and those involved in van der Waals interactions are in green circles. Hydrogen bond interactions with amino acid side chains are represented by blue dashed arrows directed toward the electron donor. Hydrogen bond interactions with amino acid main chains are represented by green dashed arrows directed toward the electron donor. Pi interactions are represented by orange lines.

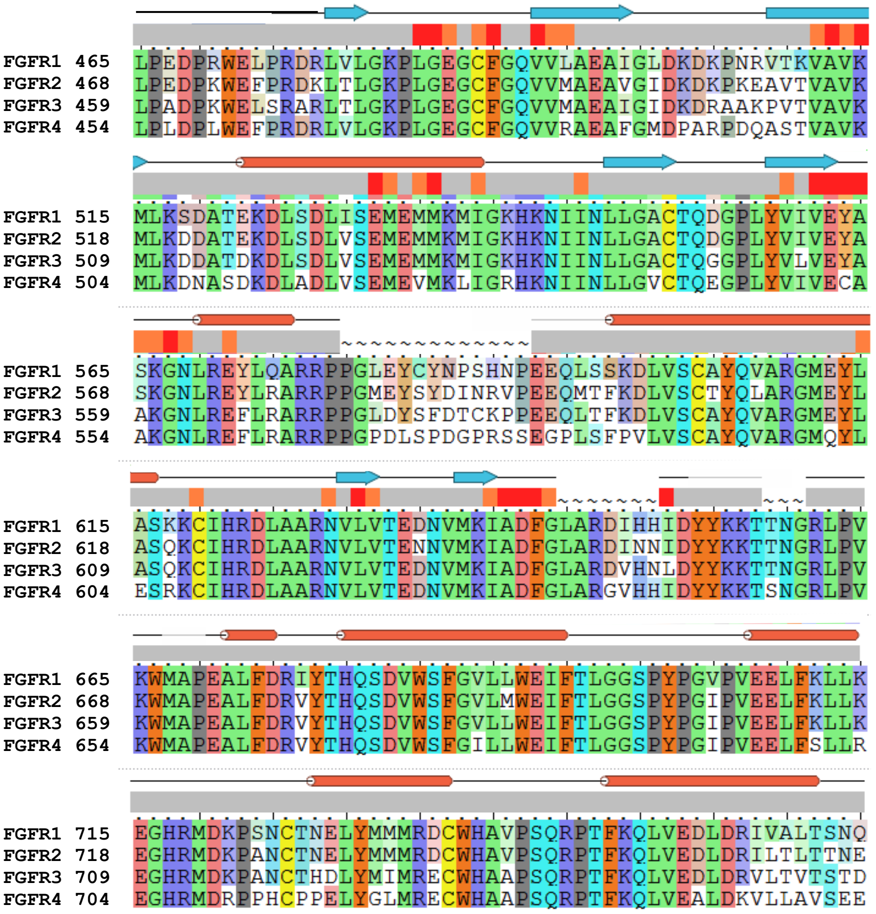

Fig. S2. Sequence alignment of FGFR1–4, secondary structural elements of FGFR1, and residue positions with shortest distance to lenvatinib.

Conserved residues are colored by residue type, and color density reflects sequence identity: hydrophobic residues are in blue, acidic in red, basic in green-yellow, and others in orange. The protein sequence identities between FGFR1 and FGFR2/ FGFR3/ FGFR4 were 87% (261/300), 83% (249/300), and 75% (225/300), respectively when the sequences indicated here were compared. Secondary structural elements in the FGFR1 crystal structure with lenvatinib are shown in schematic form above the sequence as follows: cylinder = α-helix; arrow = β-sheet; dotted line = disordered region. The second line above the sequence displays the residue positions in FGFR1 colored by the shortest distance between any heavy atom of lenvatinib and any heavy atom in the residue at that position: red (18 residues) indicates a distance of <4 Å, orange (21 residues) indicates <6 Å, and gray indicates ≥6 Å. These 18 "red" residues were highly conserved between FGFR1 and FGFR2 (100%; 18/18)/ FGFR3 (94%; 17/18)/ FGFR4 (94%; 17/18). Similarly, these 21 "orange" residues were conserved between FGFR1 and FGFR2 (100%; 21/21)/ FGFR3 (90%; 19/21)/ FGFR4 (86%; 18/21).

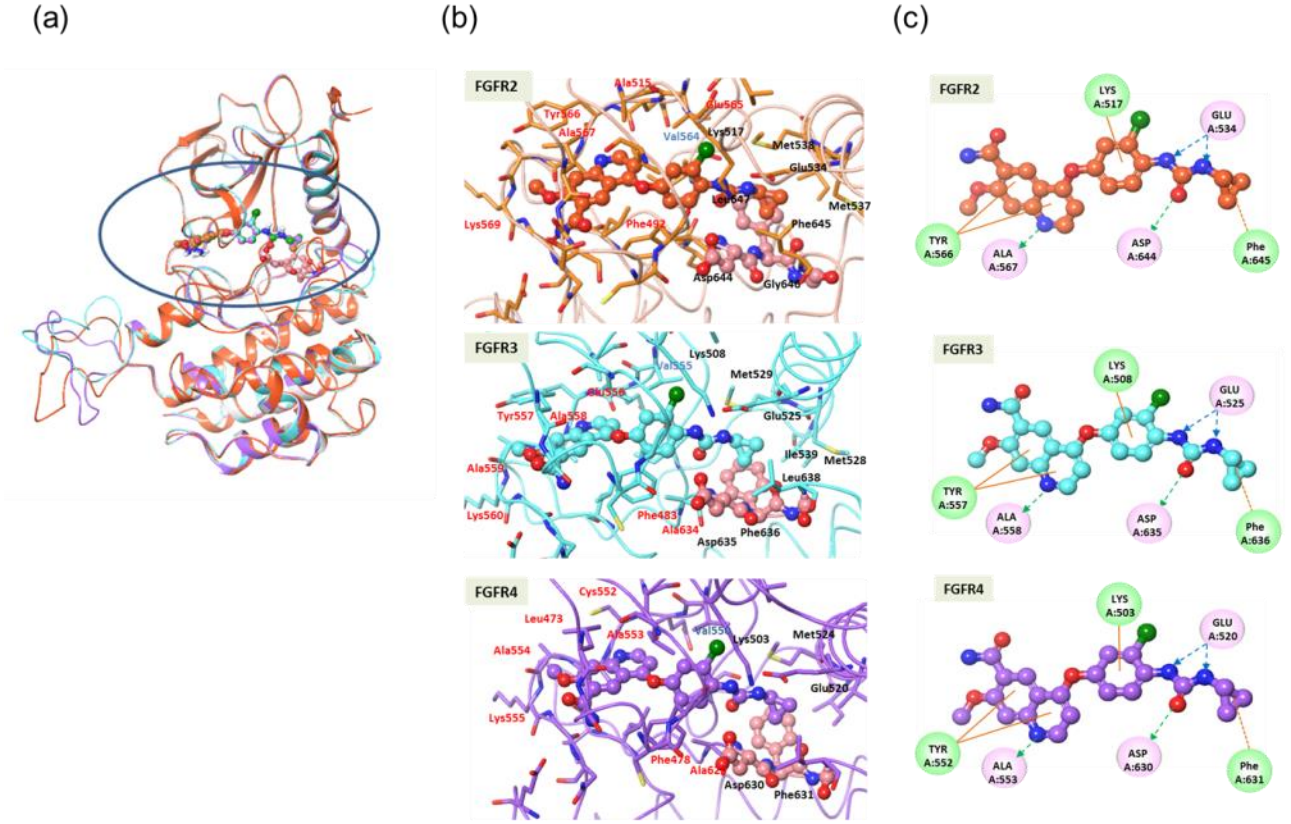

Fig. S3. Docking models of FGFR2–4 with lenvatinib.

(a) Superimposition of crystal structure (FGFR1 complex) and docking models. First ranked (i.e., most energy stable) docking models from Glide XP docking simulation using FGFR2 (orange), FGFR3 (blue), or FGFR4 (purple) homology models with lenvatinib are superimposed on the crystal structure of FGFR1. The FGFR1–lenvatinib complex is presented as a white ribbon, with C-atoms in green. In these simulations, lenvatinib bound to FGFR2, 3, and 4 in the same binding mode as it did with FGFR1. (b) Binding pocket of FGFR2–4 complex models. Lenvatinib and neighboring protein side chains are shown as stick models colored according to chemical atom type, as described above. Carbon atoms of the DFG domain are salmon colored. Residues at ATP-binding sites are indicated by red letters, and neighboring regions by black letters. The gatekeeper Val residue is indicated by blue. (c) Scheme of interaction between FGFR2–4 amino acid residues and lenvatinib observed in docking simulations. Residues that interact with ligand are circled by a different color according to the type of interaction: those involved in hydrogen bonding, charge, or polar interactions are in pink circles; and those involved in van der Waals interactions are in green circles. Hydrogen bond interactions with amino acid side chains are represented by blue dashed arrows directed toward the electron donor. Hydrogen bond interactions with amino acid main chains are represented by green dashed arrows directed toward the electron donor. Pi interactions are represented by orange lines.

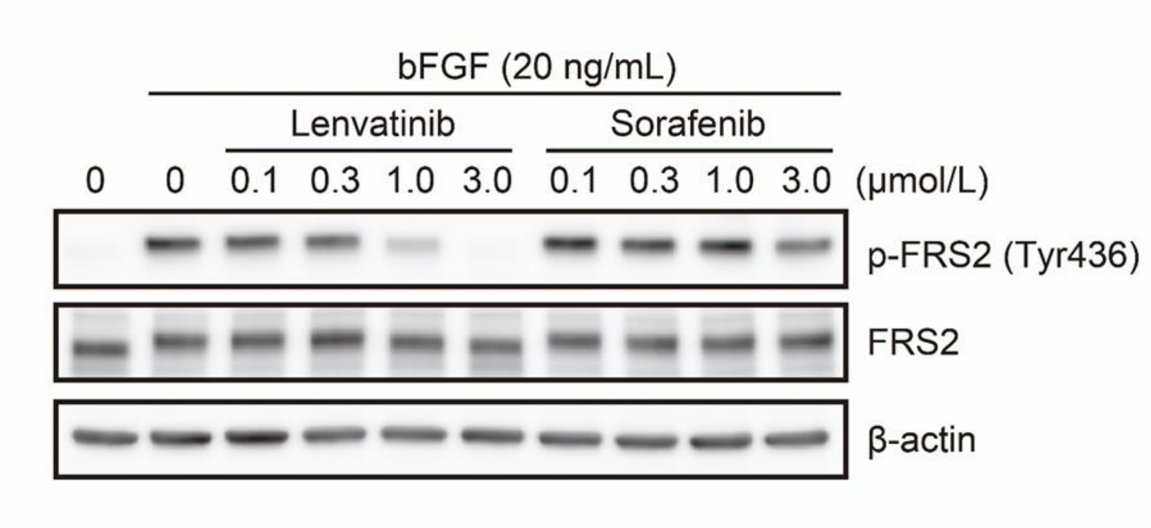

Fig. S4. Inhibitory activity of lenvatinib and sorafenib against the FGF signaling pathway in SNU-449 cells.

SNU-449 cells were treated with lenvatinib or sorafenib for 1 h, followed by bFGF stimulation (20 ng/mL) for 5 min. Cell lysates were subjected to Western blotting to detect phosphorylated FRS2, FRS2, and β-actin.

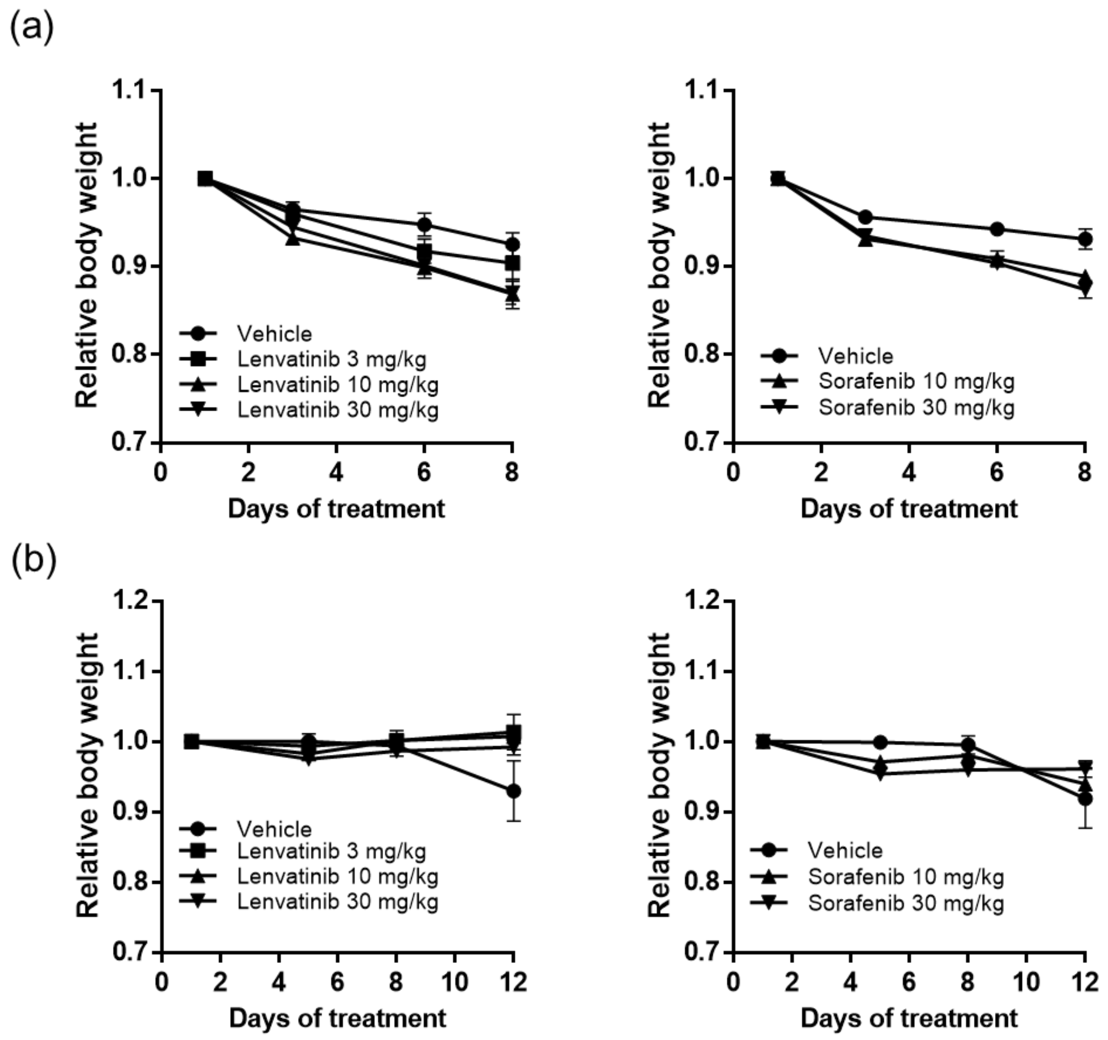

Fig. S5. Relative body weight of mice bearing Hep3B2.1-7 or SNU-398 xenografts with lenvatinib or sorafenib treatment.

(a) Hep3B2.1-7 xenograft model. (b) SNU-398 xenograft model. Mice bearing xenograft tumors were orally administered lenvatinib (3–30 mg/kg) or sorafenib (10, 30 mg/kg) or the corresponding vehicle for 7 (Hep3B2.1-7) or 11 (SNU-398) days. Data are means ± SEM (n = 8).

(a)

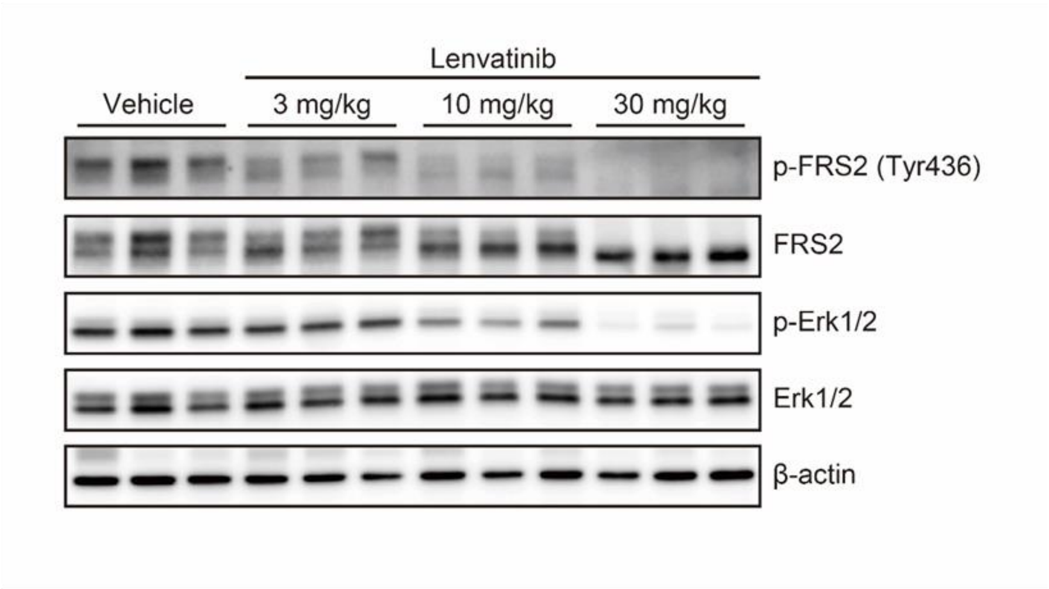

(b)

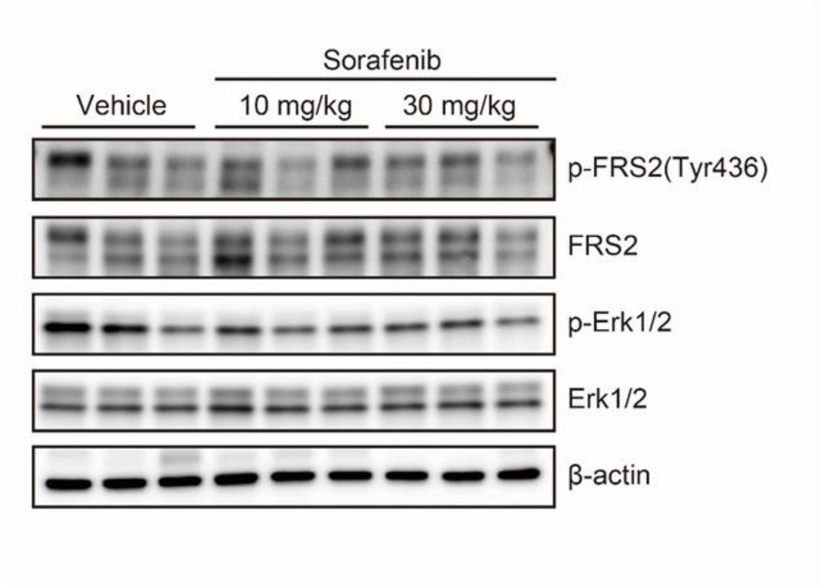

Fig. S6. Inhibitory activity of lenvatinib and sorafenib against the FGF signaling pathway in HuH-7 xenograft tumors.

Effects of lenvatinib (a) and sorafenib (b) on phosphorylation of FRS2 and Erk1/2. Tumors were collected 2 h after single treatment with lenvatinib (3–30 mg/kg), sorafenib (10, 30 mg/kg), or the corresponding vehicle control, and the tumor lysates were subjected to Western blot analysis.

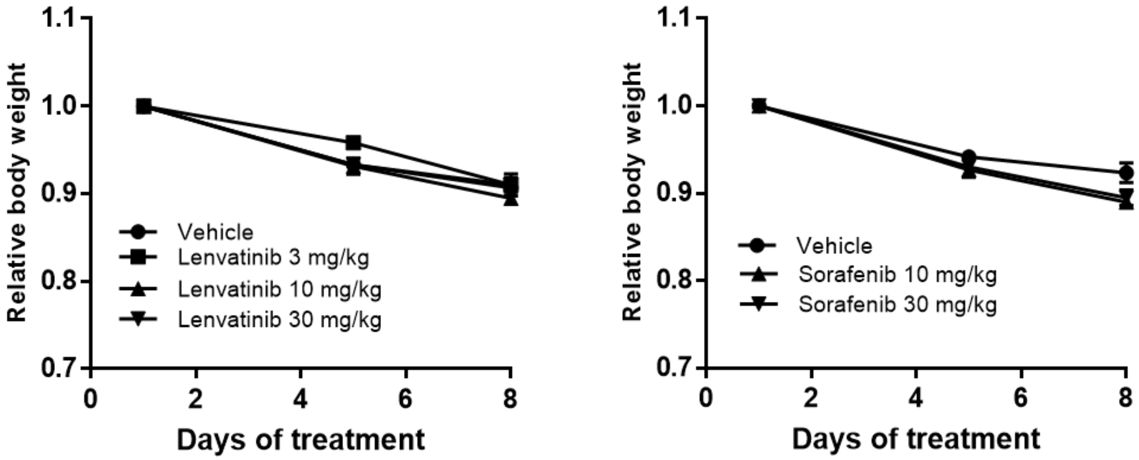

Fig. S7. Relative body weight of mice bearing PLC/PRF/5 xenografts with lenvatinib or sorafenib treatment.

Mice bearing xenograft tumors were orally administered lenvatinib (3–30 mg/kg), sorafenib (10, 30 mg/kg), or the corresponding vehicle for 7 days. Data are means ± SEM (n = 6).

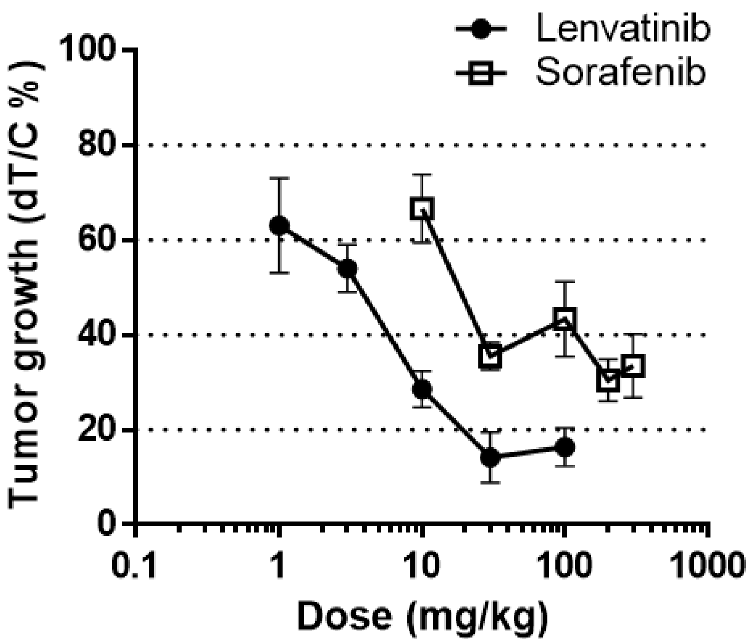

Fig. S8. Antitumor activity of lenvatinib and sorafenib in the PLC/PRF/5 xenograft model.

Mice bearing xenograft tumors were randomly allocated to groups; mice then orally received lenvatinib (1–100 mg/kg) or sorafenib (10–300 mg/kg) for 14 days. Data are presented as mean dT/C ± SEM (n = 3 to 5). dT/C (%) = dT/dC × 100, where dT and dC are increase in tumor volume from Day 1 in treated group and the corresponding vehicle control group, respectively.

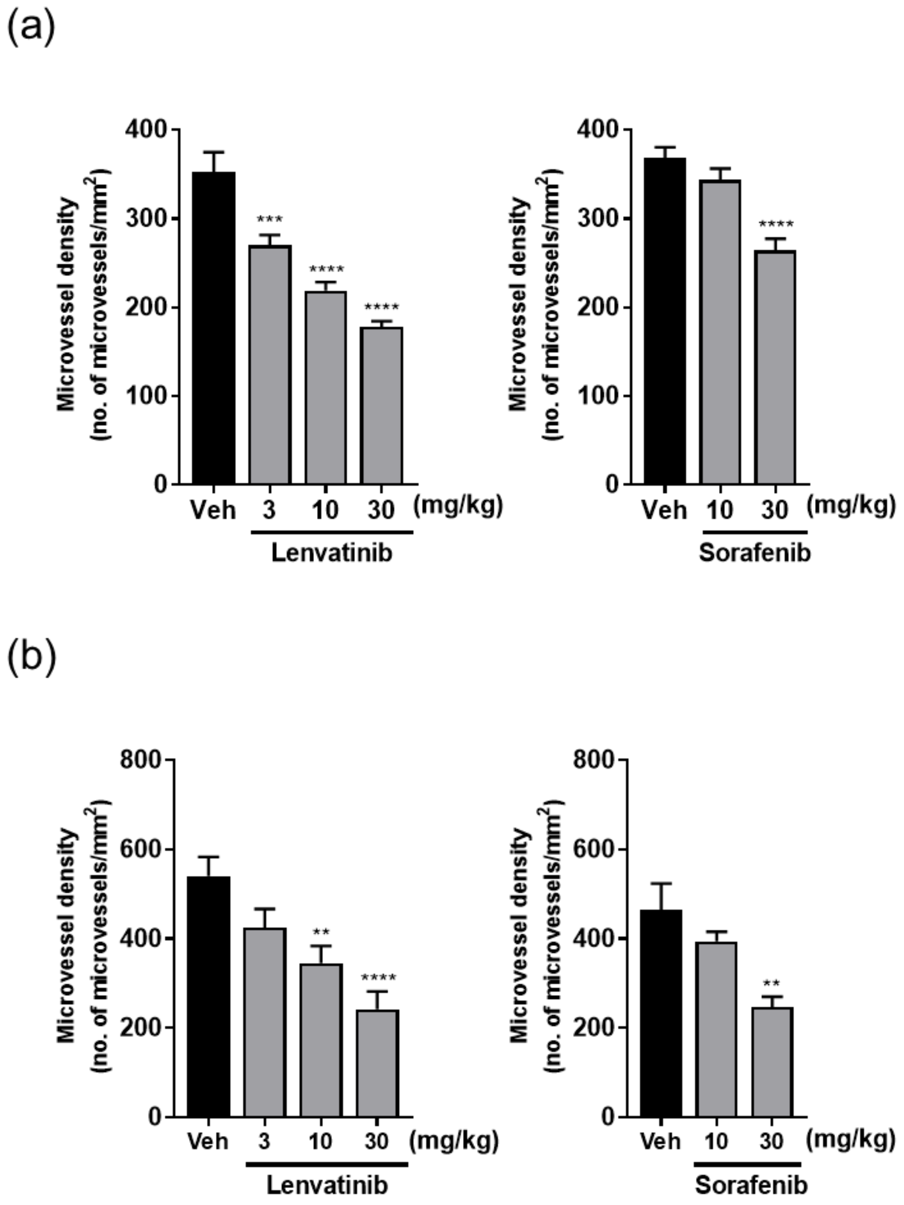

Fig. S9. Anti-angiogenic activities of lenvatinib and sorafenib in Hep3B2.1-7 and SNU-398 xenograft models.

Mice bearing xenograft tumors were orally administered lenvatinib (3–30 mg/kg), sorafenib (10, 30 mg/kg), or the corresponding vehicle (Veh) for 7 (Hep3B2.1-7) or 11 (SNU-398) days. Formalin-fixed paraffin-embedded sections of tumors were stained with anti-CD31 antibody to visualize tumor vessels, and microvessel density (MVD) was measured. (a) MVD of Hep3B2.1-7 model. (b) MVD of SNU-398 model. Data are means + SEM (n = 8). ** $P < 0.01$, *** $P < 0.001$, **** $P < 0.0001$ versus vehicle control.

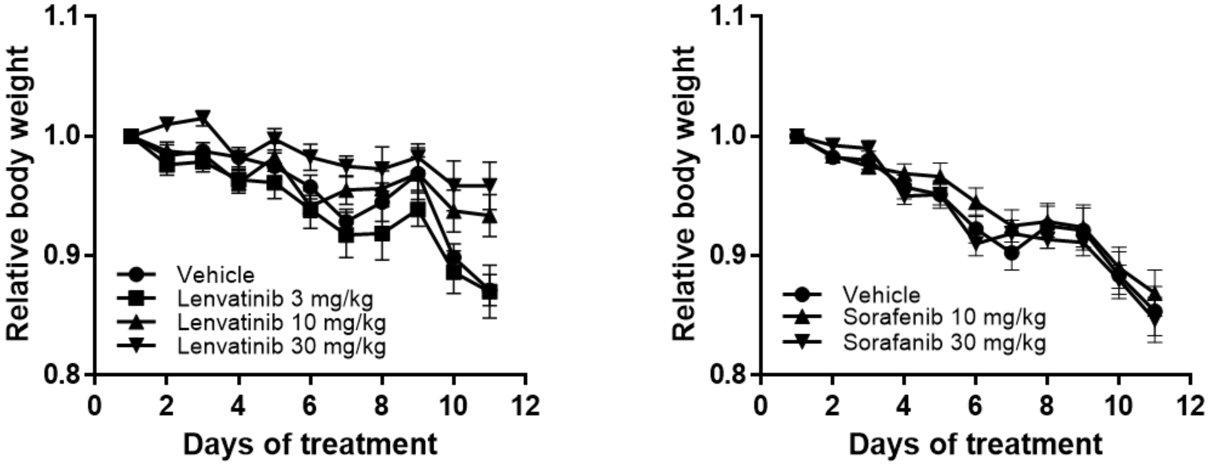

Fig. S10. Relative body weight of mice bearing PDX-derived cell line LIXC-012 xenografts with lenvatinib or sorafenib treatment.

Mice bearing xenograft tumors were orally administered lenvatinib (3–30 mg/kg) or sorafenib (10, 30 mg/kg), or the corresponding vehicle for 14 days. Data are means ± SEM (n = 8). Multiple mice in each vehicle group were removed from the study because of excess tumor volume or cachexia-induced BWL after Day 11.

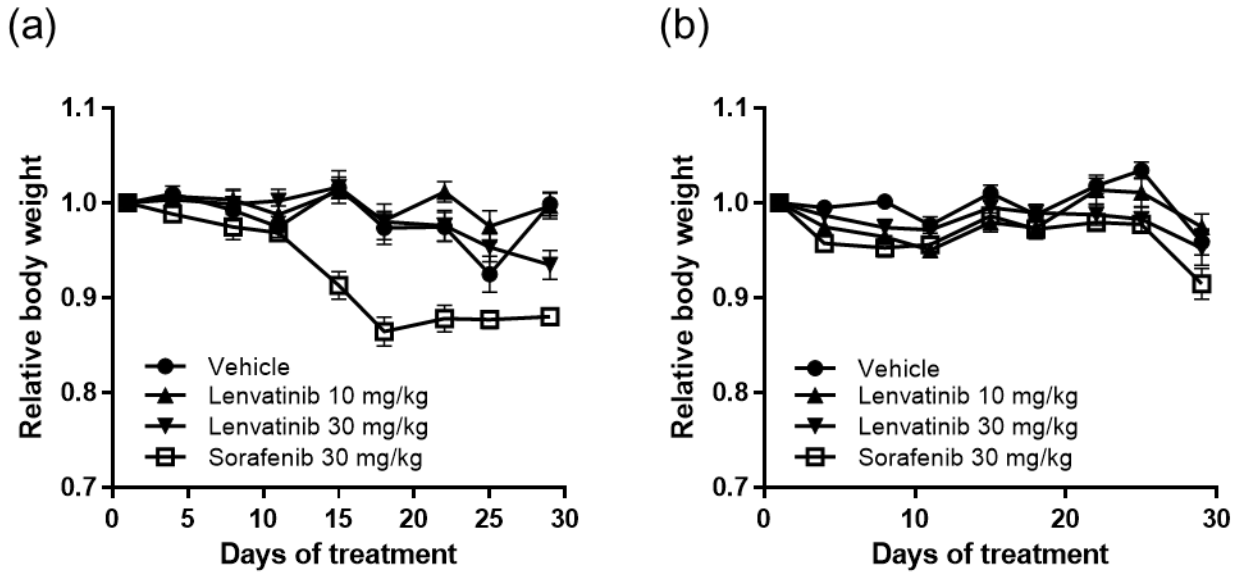

Fig. S11. Relative body weight of mice bearing HCC PDX (LI0050 or LI0334) tumors with lenvatinib or sorafenib treatment.

Mice bearing tumors were orally administered lenvatinib (10, 30 mg/kg), sorafenib (30 mg/kg), or vehicle (3 mmol/L HCl only) for 28 days. (a) LI0050 model. Sorafenib was poorly tolerated in this study, with the death of five mice (one on Day 10 and four on Day 19) and multiple dose suspensions. Dosing of one mouse in the lenvatinib (30 mg/kg) group was suspended from Day 15 to Day 27 owing to transient BWL on Day 15. Data are means ± SEM (n = 15 for vehicle and lenvatinib groups, n=10–15 for sorafenib group). (b) LI0334 model. Data are means ± SEM (n = 15).

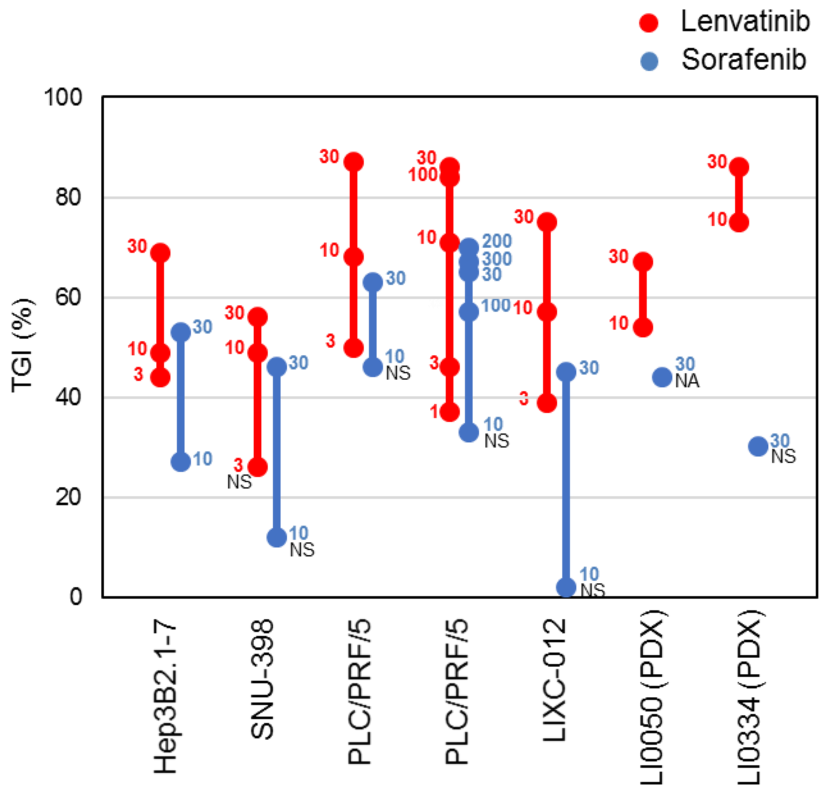

Fig. S12. Summary of tumor growth inhibition (TGI) in HCC xenograft models.

TGI (%) was calculated by using the formula: TGI (%) = 100 – [(dT/dC) × 100], where dT and dC are increases in tumor volume from Day 1 in treated group and the corresponding vehicle control group, respectively. Numbers in red or blue show the doses (mg/kg) of each treatment. NS = not significant vs. control group; NA = not applicable. Unmarked marker indicates that the TV was significantly inhibited compared with that of the corresponding vehicle control group.
