# Supplementary material for: Lenvatinib inhibits angiogenesis and tumor fibroblast growth factor signaling pathways in human hepatocellular carcinoma models
Source: Cancer Med. 2018 May 7;7(6):2641–53. doi: 10.1002/cam4.1517 (PMC6010799; doi:10.1002/cam4.1517)
Supplement: Supplementary file 2 — Table S1. Data collection and processing statistics. Table S2. Refinement statistics. Table S3. Glide docking score. [file CAM4-7-2641-s002.docx]

**Supporting Tables**

**Table S1. Data collection and processing statistics ^†^**

| X-ray source | PXI/X06SA (SLS ^‡^) |
| --- | --- |
| Wavelength [Å] | 1.0000 |
| Detector | EIGER X 16M |
| Temperature [K] | 100 |
| Space group | C2 |
| Cell: a; b; c; [Å] | 210.21; 57.46; 65.95 |
| α; β; γ; [◦] | 90.0; 106.8; 90.0 |
| Resolution [Å] | 2.86 (range, 3.11–2.86) |
| Unique reflections | 17573 (3821) |
| Multiplicity | 3.7 (3.5) |
| Completeness [%] | 99.1 (98.0) |
| R_sym_ [%] ^§^ | 4.5 (43.2) |
| R_meas_ [%] ^¶^ | 5.2 (50.6) |
| Mean(I)/sd **^††^** | 21.87 (4.18) |

**^†^** Values in parentheses refer to the highest-resolution bin.

^‡^ SWISS LIGHT SOURCE (SLS, Villigen, Switzerland).


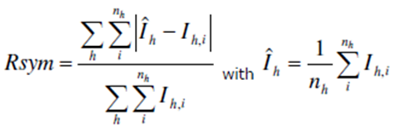


^§^

Where *I_h,j_* is the intensity value of the *i*th measurement of *h*.


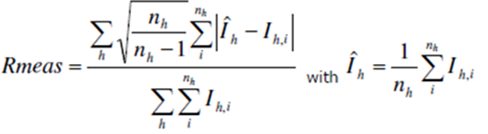


¶

Where *I_h,j_* is the intensity value of the *i*th measurement of *h*.

**^††^** Calculated from independent reflections.

**Table S2. Refinement statistics ^†^**

| Resolution [Å] | 100.65–2.86 |
| --- | --- |
| Number of reflections (working /test) | 16880 / 692 |
| R_cryst_ [%] | 21.3 |
| R_free_ [%] ^‡^ | 26.9 |
| Total number of atoms: |  |
| Protein | 4526 |
| Water | 8 |
| Ligand | 60 |
| Deviation from ideal geometry: ^§^ |  |
| Bond lengths [Å] | 0.008 |
| Bond angles [°] | 1.23 |
| Bonded Bs [Å^2^] ^¶^ | 10.7 |
| Ramachandran plot: **^††^** |  |
| Most favored regions [%] | 89.8 |
| Additional allowed regions [%] | 10.2 |
| Generously allowed regions [%] | 0.0 |
| Disallowed regions [%] | 0.0 |

**^†^** Values as defined in REFMAC5, without sigma cut-off.

^‡^ Test-set contains 3.9% of measured reflections.

^§^ Root mean square deviations from geometric target values.

¶ Calculated with MOLEMAN.

**^††^** Calculated with PROCHECK.

**Table S3. Glide docking score**

**
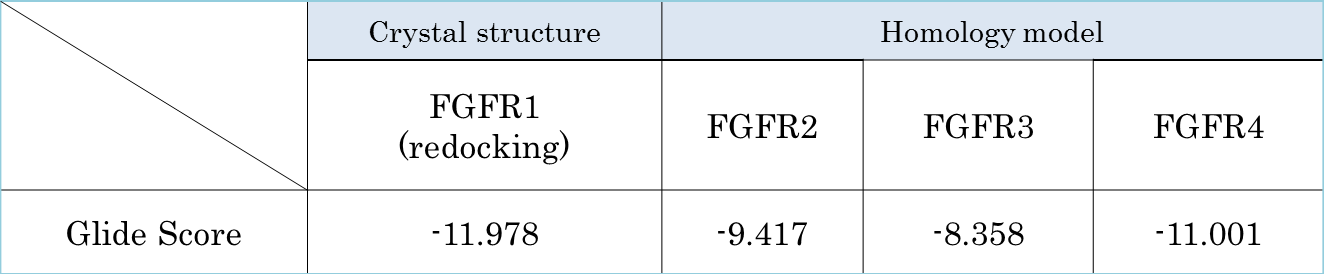
**

All docking solutions were sorted by Glide Score, and their first pose scores are listed in.

The co-crystallized lenvatinib of FGFR1 was docked against its corresponding FGFR1 structure to see whether Glide XP could predict binding poses as conformationally close to the co-crystallized pose as possible; two poses that showed almost the same docking mode with the crystal structure of lenvatinib were predicted.

From the docking simulation using FGFR2 and FGFR3 models, two solutions were obtained. From the simulation with FGFR4, one pose was calculated. All docking models showed the same binding mode for lenvatinib as that observed in the crystal complex of lenvatinib–FGFR1.
