# Supplementary material for: Lenvatinib inhibits angiogenesis and tumor fibroblast growth factor signaling pathways in human hepatocellular carcinoma models
Source: Cancer Med. 2018 May 7;7(6):2641–53. doi: 10.1002/cam4.1517 (PMC6010799; doi:10.1002/cam4.1517)
Supplement: Supplementary file 3 — Appendix S1. Supporting materials and methods. [file CAM4-7-2641-s003.docx]

**Supporting Doc. S1**

**Supporting Materials and Methods**

The following crystallographic studies (FGFR1 protein preparation, crystallization, X-ray diffraction data collection, and structure modeling/refinement of FGFR1–lenvatinib complex) were performed in Proteros biostructures GmbH.

# FGFR1 protein production and purification

Amino acids 461–774 of FGFR1 (UniProt entry P11362) containing an C488A-mutation were cloned in frame with a TEV-cleavable N-terminal HIS_6_-tag for baculovirus mediated insect cell expression. The protein was expressed in Sf9 cells and purified for crystallization by using a three-step chromatography procedure on NiNTA (Qiagen, Hilden, Germany), TEV-cleavage followed by a second pass over NiNTA, and a final gel filtration step on a Superdex200 26/60 column (GE Healthcare Life Sciences, Little Chalfont, UK). Pure protein was pooled after SDS-PAGE analysis and concentrated to ~11 mg/mL in 10 mmol/L Tris-HCl (pH 8.0), 10 mmol/L NaCl, 2 mmol/L DTT.

# Crystallization of lenvatinib–FGFR1 complex and X-ray diffraction data collection

Purified FGFR1 was used at a concentration of 10 mg/mL. Hanging drop vapor diffusion crystallization drops were set-up by mixing 500 nL FGFR1 with 500 nL reservoir solution (17.7% PEG [polyethylene glycol] 5000 MME [monomethyl ether], 0.2 mol/L (NH_4_)_2_ SO_4_, 0.1 mol/L Tris-HCl [pH 7.0]) and incubated at 277K. Single apo-crystals of FGFR1 were transferred to a soaking drop that contained 90% reservoir solution + 10% lenvatinib (100 mmol/L DMSO stock solution, final ligand concentration 10 mmol/L). After 7 h incubation the crystals were transferred to a cryo-solution (65% reservoir solution, 10% lenvatinib and 25% ethylene glycol) and immediately flash frozen in liquid nitrogen.　X-ray diffraction data were collected from complexed crystals of FGFR1 at the Swiss Light Source (SLS; Villigen, Switzerland) under cryogenic conditions (100K). The crystal belonged to space group C2. All data sets were processed with XDS and XSCALE (Table S1)^1^.

# FGFR1–lenvatinib structure modeling and refinement

The phase information necessary to determine and analyze the structure was obtained by molecular replacement. A previously solved structure of FGFR1 was used as a search model. Subsequent model building and refinement were performed according to standard protocols with the software packages CCP4 (Version 6.2.0)^2^ and COOT (Version 0.6)^3^. To calculate the free R-factor—a measure for cross-validating the correctness of the final model—about 3.9% of measured reflections were excluded from the refinement procedure (Table S2).

Automatically generated local NCS restraints have been applied (keyword “ncsr local” of newer REFMAC5 versions)^4^. Ligand parameterization and generation of the corresponding library files were performed with the program CORINA (Molecular Networks GmbH Computerchemie, Erlangen, Germany).

The water model was built with the “Find waters” algorithm of COOT by putting water molecules in peaks of the Fo-Fc map contoured at 3.0; this was followed by refinement with REFMAC5 and checking of all water molecules with the validation tool of COOT. The occupancy of side chains, which were in negative peaks in the Fo-Fc map (contoured at –3.0 σ), was set to zero. Subsequently, it was set to 0.5 if a positive peak occurred after the next refinement cycle.

Statistics of the final structure and the refinement process are listed in Table S2.

# Protein structure preparation by homology modeling and docking simulation

Maestro ver.11.2 software (Schrödinger, LLC, New York, NY) was used in homology modeling and docking simulations. Homology models of the kinase domain of human FGFR2, -3 and -4 were built by using the Prime^5^ modeling program with FGFR1 in complex coordinates with lenvatinib as the template structure, according to the sequence alignments shown in Figure S2. Hydrogen atoms were added, and a brief relaxation was performed on each starting structure by using the Protein Preparation Wizard^6^. The binding models of each FGFR model with lenvatinib were modeled by using XP (extra-precision) Glide^7^ with OPLS3 force field^8^.

# Western blotting (SNU-449)

SNU-449 cells were starved overnight in culture medium containing 0.5% BSA; incubated with vehicle (DMSO), lenvatinib, or sorafenib for 1 h; treated with 20 ng/mL basic-FGF (bFGF; Thermo Fisher Scientific, Waltham, MA, USA) for 5 min; and then washed and lysed with RIPA buffer containing protease inhibitor cocktail (Roche Diagnostics, Mannheim, Germany) and Halt phosphatase inhibitor cocktail (Thermo Fisher Scientific, Waltham, MA, USA). SDS-PAGE and band visualization were performed as described in the Materials and Methods.

# PLC/PRF/5 xenograft model treated with higher doses of compounds

Female BALB/c nude mice were inoculated subcutaneously in the right flank with PLC/PRF/5 cells (3.5 × 10^6^) in PBS mixed with Matrigel [1:1] (Corning, Corning, NY, USA). Once the tumors reached approximately 540 mm^3^, the mice were randomly allocated to treatment groups (Day 1). Lenvatinib (1–100 mg/kg), sorefenib (10–300 mg/kg), or the corresponding vehicle solution was given orally to individual mice once daily for 14 days. Tumor growth (dT/C [%]) was calculated by using the formula: dT/C (%) = dT/dC × 100, where dT and dC are increases in tumor volume from Day 1 in treated group and the corresponding vehicle control group, respectively.

# References

[1] Kabsch W. XDS. Acta Crystallogr D Biol Crystallogr. 2010; 66: 125-32.

[2] The CCP4 suite: programs for protein crystallography. Acta Crystallogr D Biol Crystallogr. 1994; 50: 760-3.

[3] Emsley P, Cowtan K. Coot: model-building tools for molecular graphics. Acta Crystallogr D Biol Crystallogr. 2004; 60: 2126-32.

[4] Murshudov GN, Vagin AA, Dodson EJ. Refinement of macromolecular structures by the maximum-likelihood method. Acta Crystallogr D Biol Crystallogr. 1997; 53: 240-55.

[5] Jacobson MP, Pincus DL, Rapp CS, et al. A hierarchical approach to all-atom protein loop prediction. Proteins. 2004; 55: 351-67.

[6] Sastry GM, Adzhigirey M, Day T, Annabhimoju R, Sherman W. Protein and ligand preparation: parameters, protocols, and influence on virtual screening enrichments. J Comput Aided Mol Des. 2013; 27: 221-34.

[7] Friesner RA, Murphy RB, Repasky MP, et al. Extra precision glide: docking and scoring incorporating a model of hydrophobic enclosure for protein-ligand complexes. J Med Chem. 2006; 49: 6177-96.

[8] Harder E, Damm W, Maple J, et al. OPLS3: A Force Field Providing Broad Coverage of Drug-like Small Molecules and Proteins. J Chem Theory Comput. 2016; 12: 281-96.
